# Should AI models be explainable to clinicians?

**DOI:** 10.1186/s13054-024-05005-y

**Published:** 2024-09-12

**Authors:** Gwénolé Abgrall, Andre L. Holder, Zaineb Chelly Dagdia, Karine Zeitouni, Xavier Monnet

**Affiliations:** 1 AP-HP, Service de Médecine Intensive-Réanimation, Hôpital de Bicêtre, DMU 4 CORREVE, Inserm UMR S_999, FHU SEPSIS, CARMAS, Université Paris-Saclay, 78 Rue du Général Leclerc, 94270 Le Kremlin-Bicêtre, France; 2 Service de Médecine Intensive Réanimation, Centre Hospitalier Universitaire Grenoble Alpes, Av. des Maquis du Grésivaudan, 38700 La Tronche, France; 3 Division of Pulmonary, Critical Care, Allergy and Sleep Medicine, Department of Medicine, Emory University School of Medicine, Atlanta, GA USA; 4 Laboratoire DAVID, Université Versailles Saint-Quentin-en-Yvelines, 78035 Versailles, France

**Keywords:** Explainable artificial intelligence, Interpretability, Clinical decision-making, Regulatory compliance, Algorithmic bias, Patient autonomy, Fairness, Transparency, Generative artificial intelligence

## Abstract

In the high-stakes realm of critical care, where daily decisions are crucial and clear communication is paramount, comprehending the rationale behind Artificial Intelligence (AI)-driven decisions appears essential. While AI has the potential to improve decision-making, its complexity can hinder comprehension and adherence to its recommendations. “Explainable AI” (XAI) aims to bridge this gap, enhancing confidence among patients and doctors. It also helps to meet regulatory transparency requirements, offers actionable insights, and promotes fairness and safety. Yet, defining explainability and standardising assessments are ongoing challenges and balancing performance and explainability can be needed, even if XAI is a growing field.

**Supplementary Information:**

The online version contains supplementary material available at 10.1186/s13054-024-05005-y.

## Introduction

The healthcare sector has witnessed a surge in Artificial Intelligence (AI) models, particularly in crucial areas such as medical imaging, perioperative, and critical care, where extensive volumes of data are constantly generated. In these fields, the rapid development of AI-based models holds significant potential for enhancing medical decision-making and improving patient outcomes [1].

However, a recent survey of intensive care unit (ICU) professionals sheds light on their doubts regarding AI [2]. Seventy-one percent of participants were either unsure or disagreed that AI can be used reliably in ICU decision-making. The usual diffidence in a novelty may be at least partially responsible. However, this lack of confidence could also come from distrust of decisions based on algorithms that resemble "black boxes". This prompts the question: should AI models be made explainable to clinicians?

## Background

The AI literature offers varied interpretations of explainability, underscoring the absence of a formal definition. Sometimes, explainability is mistakenly used interchangeably with interpretability and transparency [3]. Interpretability may refer to the degree to which a human can understand the internal mechanisms and decision-making processes of an AI model [4]. Interpretable models are designed to be easily understood and straightforward, enabling users to trace and grasp how inputs are transformed into outputs, sometimes through an identifiable pathophysiologic rationale. Examples of inherently interpretable models include decision trees and linear regression, where the logic and rules governing the model's decisions are clear and easy to follow.

Explainability, in contrast, involves techniques and methods used to make the decisions of more complex, often opaque models (like deep neural networks) understandable to humans. This typically involves post hoc explanations, which are generated after the model has made its decisions. Hence, techniques such as Local Interpretable Model-agnostic Explanations (LIME) and Shapley Additive Explanations (SHAP) are commonly used to clarify which factors influenced the model’s predictions and why they did so, without necessarily simplifying the model itself or understanding the underlying biochemical mechanism (See Additional file 1).

## Models should be explainable for clinicians: yes!

### A right to explanation?

The European General Data Protection Regulation (GDPR) requires that individuals be informed about automated decision-making processes. This includes their underlying mechanisms, significance, and potential consequences of their application for the individual. The information provided should be sufficiently comprehensive to ensure the understanding of the decision's rationale and, potentially, their right to challenge the algorithm's outcomes (Articles 13, 14, 15, 22 and Recital 71 [5, 6]).

While the GDPR does not explicitly define a "right to explanation," some experts interpret these requirements as effectively establishing one [7]. Nonetheless, there is considerable debate about the extent to which the regulation genuinely provides this right [8–10].

The recent Artificial Intelligence Act emphasises the necessity of transparency and human oversight in high-risk AI systems. Specifically, it mandates that these systems—including many AI-powered medical devices [11]—must be designed and developed to ensure "sufficient transparency to enable users to interpret the system’s output" and "use it appropriately" (Article 13 [12]). This emphasis on transparency aims to build trust and accountability by making AI systems understandable and open to scrutiny. However, the Act does not provide specific level for explainability [10].

### Facilitating AI acceptance in decision-making

In the high-pressure environment of the ICU, doctors need clarity when making decisions, especially when using AI-based support systems [13]. The lack of transparency in AI models can impede trust in their diagnostic, therapeutic, and prognostic suggestions, leading to potential "decision paralysis". This is further exacerbated by accountability concerns: how can one take responsibility for decisions based on AI models that are not fully understood [14]?

Critical care doctors frequently encounter syndrome-based diseases, such as acute kidney injury (AKI) and sepsis, as well as events like the need for mechanical support, all marked by notable heterogeneity. Their partially understood nature poses challenges for many AI models to promptly identify effective interventions for treatment or prevention. Explainable AI (XAI) models can be more actionable (for definitions and descriptions of explainable AI (XAI) terminology, consult the Additional file 1). For instance, the models developed by Lauritsen et al. [15], provide early warnings for various critical illnesses, while pinpointing the specific factors driving their predictions for each patient. These models not only offer state-of-the-art, real-time predictions for critical illnesses like sepsis, AKI, or acute lung injury (ALI), but also provide insights into the electronic health records underpinning these predictions that would otherwise have remained unidentified. Such an approach enables practitioners to respond more effectively and personally, focusing on modifiable factors.

From a patient's perspective, the opacity of AI systems can also impair their comprehension, impacting their informed consent and autonomy. This lack of clarity could unintentionally shift decision-making power from patients and doctors to less transparent algorithms, potentially fostering a new kind of medical paternalism where it is assumed that "computers know the best" [16]. To navigate these challenges effectively, clinicians could benefit from understanding the rationale behind the outcomes produced by AI-based models. The paramount focus should be on deciphering why a particular AI model arrives at specific results and the underlying factors influencing its decision-making process. This approach parallels the collaborative mental models that clinicians establish with their colleagues, akin to seeking a second opinion [17]. In addition, with this knowledge in hand, clinicians should communicate more effectively with patients and their families, facilitating informed decisions about their healthcare [18].

### Ensuring safety, clinical relevance, and fairness

Engineers and clinicians have distinct expectations about model explainability. Engineers typically focus on the interpretability of the model’s inner workings, such as for debugging purposes, whereas clinicians emphasise the clinical relevance of its outputs [19]. Hence, drawing a parallel with their role in pharmacovigilance, clinicians should play a central role in evaluating AI models throughout their lifecycle.

In this context, explainable models may help identifying spurious correlations that could lead to iatrogenic harm. For example, Deasy et al. [20] proposed an AI model that predicts in-hospital mortality for ICU patients using numerous variables derived from the MIMIC-III database [21], a comprehensive collection of critical care data, without prior variable selection. A closer look into its functioning revealed that certain features, such as a priest's visit, were strong predictors of imminent mortality. In a scenario where this model is applied practically, if religious visit patterns change, the model might wrongly predict how likely patients are to survive. This could cause medical teams to either act too slowly or take unnecessary actions.

Similarly, during the COVID-19 pandemic, researchers harnessed AI-driven models to analyse X-rays and CT scans for quick identification of COVID-related pneumonia. DeGrave et al. used post-hoc explainability methods such as saliency maps and generative adversarial networks (GANs) to study their trustworthiness. Saliency maps highlight the most influential image regions for model predictions, while GANs transform images to reveal key features differentiating classes (See Additional file 1). They demonstrated that some deep-learning models took 'shortcuts' by relying on features like laterality markers (e.g., the "R" letter adjacent to the right side of the radiograph) or patient positioning to draw their conclusions, rather than focusing on medically relevant pathology [22], rendering their predictions less reliable.

To ensure the transparent use of AI in healthcare, a thorough examination of potential biases and disparities arising from the inclusion or exclusion of certain variables is essential. An important example is the historical use of racial or ethnic data in calculations of glomerular filtration rates, a practice that has led to increased diagnostic disparities in kidney disease among marginalised groups [23]. Consequently, when AI is used for purposes such as predicting AKI, it is imperative for clinicians to clearly understand how the algorithm incorporates sensitive demographic data. They need to be keenly aware of the effects of such data on both the accuracy and fairness of the model's predictions, in order to avoid reinforcing existing healthcare inequalities [24]. This is not only ethically prudent, but in some instances has become a governmental priority [25].

## Models should be explainable for clinicians: no!

### The proof is in the pudding?

When a model has no significant impact or has proven its performance sufficiently, the cost of explanation may outweigh the benefit [26]. If an AI model consistently outperforms a clinician, even without being explainable, it could be considered ethically justifiable to use it. In such cases, employing the AI as a co-pilot becomes a viable option, provided the clinician can independently verify and confirm the accuracy of the AI's decisions [27].

It is sometimes suggested that there may be a trade-off between accuracy and explainability when incorporating an explanation mechanism in AI systems [28]. A study [29] found that in medical scenarios (e.g., stroke diagnosis), the general public prioritised accuracy over explainability, emphasising the need for accurate and timely decisions for better outcomes. Conversely, in non-healthcare scenarios (e.g., criminal justice), explainability was valued more for ensuring fairness and transparency. Although post-hoc explainability can help mitigate the trade-off between accuracy and explainability, the difference in priorities across different sectors of society underscores the need for context-specific AI policy development and public engagement.

Likewise, it can be argued that even in intensive care, especially in predictive models, there are areas where understanding the associations behind an algorithm matters less than its efficiency and promptness. For instance, the Hypotension Prediction Index (HPI) from Edwards Lifesciences Corp. (Irvine, USA) uses a machine learning algorithm to forecast hypotension by analysing physiological alterations in the artery waveform. By employing variables selected from millions of individual and combinatorial ones, derived from invasive arterial line waveform analysis, it efficiently predicts and prevents intraoperative hypotension, despite lacking a straightforward physiological explanation for its output [30, 31].

### Is explainability reliable?

**Fig. 1:**
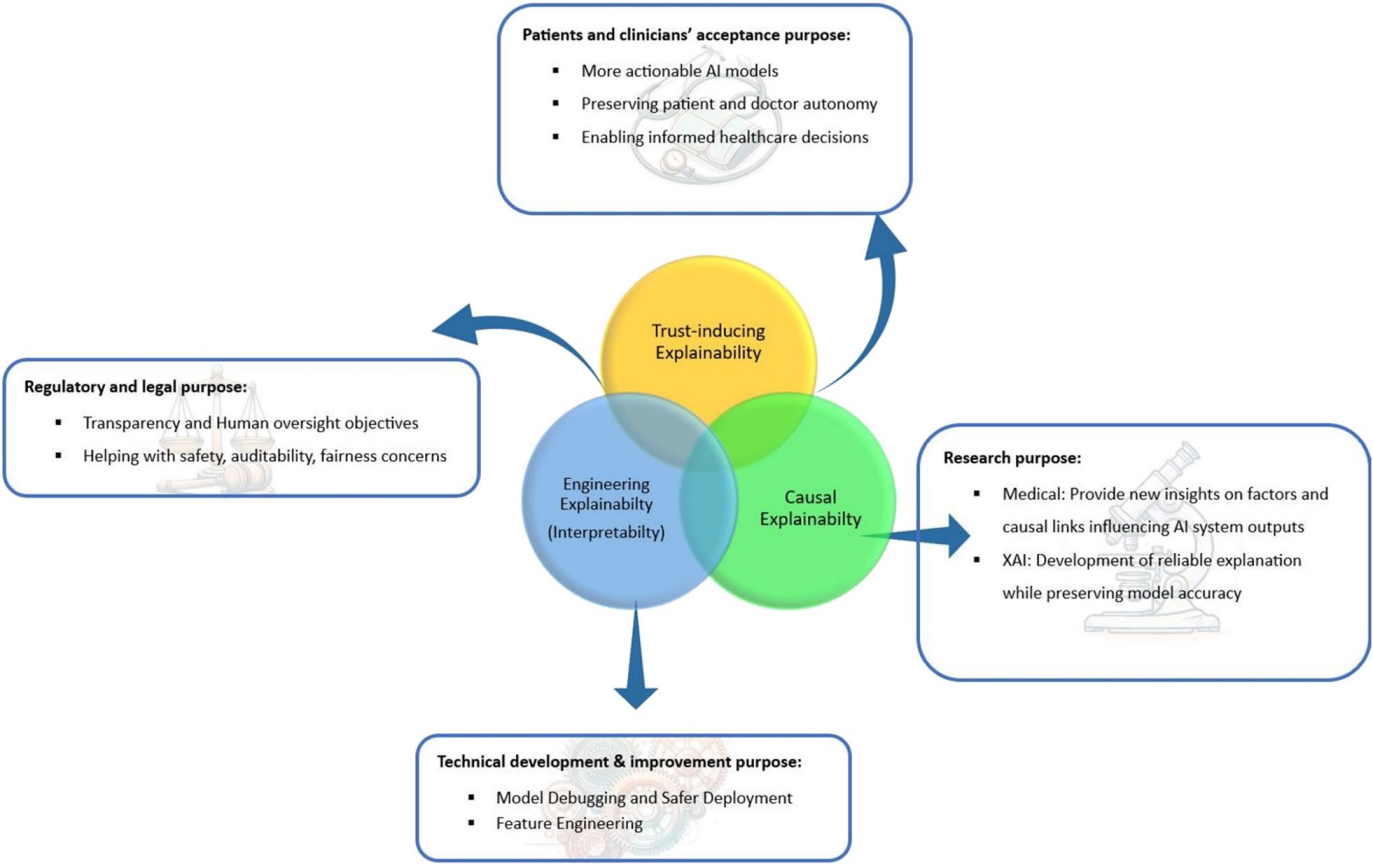
Which explainability for which audience?

Explainability, as previously mentioned, can have multiple meanings, and can vary according to stakeholders’ unique expectations (Fig. 1). Additionally, numerous XAI methods exist (Additional file 1: Fig. S1), yet standardised methods for assessing their accuracy and comprehensiveness are deficient [32, 33].

Even state-of-the-art XAI methods often provide erroneous, misleading, or incomplete explanations, especially as the complexity of models increases [10]. For example, post-hoc methods, which use external tools to clarify an algorithm's operations often without deeply examining its core workings, are inherently prone to approximations [34]. When attempting to emulate the predictions of black-box models, they might rely on different features for their explanations, potentially leading to a misinterpretation of the model's true processes. Moreover, identifying an AI model's key features does not ensure their effective or expected use, particularly from a clinical perspective [28]. For instance, saliency maps can indicate where the model is “looking,” but not what the model actually “sees” [34].

These caveats partly explain why there is still no consensus on whether AI models, as seen in decision support systems, should inherently possess explainability as a core attribute [35]. In this context, recent advancements in generative AI, such as OpenAI's ChatGPT, present significant challenges to reliable explainability. These challenges notably include model complexity, limited access to the internal workings of proprietary systems, and the difficulty of evaluating explanations without clear benchmarks [36].

### Recognising our own cognitive biases

We should recognise the ubiquity of black boxes in various domains. In many medical practices, clinicians commonly use numerous medications such as paracetamol, as well as diagnostic tools like as lab tests and magnetic resonance imaging (MRI), without fully understanding their inner workings. This prevailing lack of transparency mirrors the concept of black boxes, where the intricacies of interventions remain elusive. Similarly, the human body remains an enigma in many respects [37].

Furthermore, human clinicians are often not held to the same stringent standards of explainability as AI systems [38]. Everyday crucial decisions made by an intensivist, such as admitting patients to the intensive care unit, often involve elements of inexplicability due to intuition or implicit biases [39]. AI systems, on the other hand, can be held to a higher standard of explainability, which may not always be realistic or necessary. This double standard has led some authors to argue that the explainability requirements for AI should be considered relative to those of human decision-makers [40] for a fair and practical evaluation of decision-making in medical contexts.

Healthcare practitioners might place unwarranted confidence in models that highlight explainability. In fact, when using these models, their capacity to identify and correct major model errors seems reduced. Authors have suggested that this overconfidence may, in part, arise from an "information overload" effect [32, 41], which might also induce data fatigue.

Similarly, it is essential not to consider the workings of AI models strictly through an "anthropomorphic" perspective or to insist on just causal explanations. AI models can integrate factors that significantly improve predictive accuracy, even if these factors do not have a clear causal link to the model's outcomes [42]. While it is vital to steer clear of spurious correlations, it is worth noting that not all diseases are entirely understood in causal terms. Some might be influenced by unpredictable external factors, rather than being purely deterministic.

### From explainable AI to trustworthy AI

Ensuring the trustworthiness of AI systems is essential for promoting their widespread adoption in high-stakes ICU environments and for their routine use in decision-making. While explainability plays a role, it is neither fully sufficient [43] nor strictly indispensable for cultivating acceptance of AI systems. Trust does not arise merely from meeting a single criterion; it emerges from a combination of AI system attributes, including reliability, safety, fairness, and auditability [44]. These principles should act as a framework for evaluating AI systems throughout various stages of their lifecycle, from data collection and preprocessing to model training, evaluation, and deployment [3, 45].

Transparency of AI systems, as advocated by regulations, appears here as a cornerstone to foster trust in AI technologies. In a holistic approach to system opacity, it refers to the degree to which appropriate information about a device—including its intended use, development, performance, and underlying logic—is clearly communicated to stakeholders. [46]. The recent AI Act emphasises the need for transparency and human oversight in high-risk AI systems. Instead of mandating the use of XAI tools, it ensures users receive pertinent documentation and information [47].

In the ICU context, this information could be presented via user-friendly graphical interfaces, complemented by a robust documentation approach. This could include "model facts" sheets [37], specifically designed to provide essential model information to clinical end users. Table 1 summarises the essential aspects clinicians need to focus on when implementing AI Models in the healthcare environment.

**Table 1:** Top 10 must-knows for clinicians using AI models

1. *Objective & Scope*
Purpose: Model’s primary goal (e.g., prediction, diagnosis, recommendation)
Target population: The patient demographic the model caters to
2. *Model insights*
Structure: A concise description of the model's design
Explainability: Clarity of the model outputs for clinicians and patients
Key variables: Main features the model use, and their medical relevance
3. *Data source*
Data origin: Where training and validation data comes from, ensuring relevance to clinician's patient base
Adaptability: Ability to retrain the model using local datasets
Open access: Accessibility to data/code for replication (e.g., on platforms like GitHub)
4. *Evaluation & Validation*
Performance metrics: Measures of model accuracy
Benchmarking: Comparison to simpler, more interpretable models
Practical validation: Testing in real clinical settings, beyond just retrospective data
5. *Model limitations*
Performance concerns: Situations or conditions where model efficacy may diminish
Reliability: Model’s expression of confidence and uncertainty in its results
Error management: Approaches for handling and correcting inaccurate outputs
6. *Clinical integration*
Human oversight: Human involvement in model-driven decisions
Workflow integration: Model's fit into existing clinical processes
User experience: Interface design and clarity of information
Training & education: learning resources provided for staff and clinicians
7. *Ethical considerations*
Demographic equity: Performance consistency across diverse patient groups
Fairness audit: Efforts to identify and rectify potential biases
8. *Regulatory aspects*
Data privacy & security: Protocols for patient data management and protection
Legal adherence: Compliance with regulations like GDPR, AI Act
Clinician liability: Responsibilities when using the model
9. *Maintenance & Audit*
Safety checks: Monitoring model safety and efficiency
Updates & evolution: Keeping the model current line with new data and insights
10. *Feedback & Reporting*
Feedback channels: Systems for collecting and addressing user feedback
Adverse event: Procedures to handle and report any negative outcomes associated with the model's deployment

## Conclusion

Over the past decade, research in AI and machine learning applications in medicine has witnessed an impressive 20-fold increase [48]. However, the practical integration of these advanced methodologies into healthcare can be hindered by trust issues [19]. Increased transparency is deemed essential, and explainability is considered a crucial component of this endeavour, even though questions persist about determining the appropriate level of explainability for a specific audience (Fig. 1). This implies facing challenges across legal, ethical, technical, and economic dimensions [47].

The notion that a necessary trade-off exists between accuracy and explainability in AI models is being re-evaluated with the expansion of the field of XAI research [34, 49, 50]; (Additional file 1: Fig. S2). In medical AI, where models are typically based on detailed, structured data grounded in physiopathology, the performance difference between interpretable and more complex models often turns out to be minimal [34].

However, explainability alone does not guarantee effective AI application. It remains pivotal to grasp the implications of employing AI models, as well as to understand when and how to integrate them into clinical judgement while preserving patient autonomy in shared decision-making [16].

## Supplementary Information

**Additional file 1: Fig. S1.** This document provides a comprehensive overview of explainable artificial intelligence (XAI), detailing definitions, the difference between explainability and interpretability, and the classification of explanations as global or local. It includes a taxonomy of XAI methods. **Fig. S2.** It also addresses the balance between model complexity and the necessity for explainability in healthcare.

## Data Availability

Not applicable.

## Abbreviations

AI: Artificial Intelligence
AKI: Acute Kidney Injury
ALI: Acute Lung Injury
ARDS: Acute Respiratory Distress Syndrome
CT: Computed Tomography
GANs: Generative adversarial networks
GDPR: General Data Protection Regulation
HPI: Hypotension Prediction Index
ICU: Intensive Care Unit
LIME: Local Interpretable Model-agnostic Explanations
MIMIC: Medical Information Mart for Intensive Care
MRI: Magnetic Resonance Imaging
SHAPE: Shapley Additive Explanations
USA: United States of America
XAI: Explainable AI
