# Supplementary material for: Should AI models be explainable to clinicians?
Source: Crit Care. 2024 Sep 12;28:301. doi: 10.1186/s13054-024-05005-y (PMC11391805; doi:10.1186/s13054-024-05005-y)
Supplement: Supplementary file 1 — Additional file 1: Fig. S1. This document provides a comprehensive overview of explainable artificial intelligence (XAI), detailing definitions, the difference between explainability and interpretability, and the classification of explanations as global or local. It includes a taxonomy of XAI methods. Fig. S2. It also addresses the balance between model complexity and the necessity for explainability in healthcare. [file 13054_2024_5005_MOESM1_ESM.pdf]

Should AI models be explainable to clinicians?

By

Gwénolé ABGRALL[(1)(2)]

Andre L. HOLDER[(3)]

Zaineb CHELLY DAGDIA [(4)]

Karine ZEITOUNI[(4)]

Xavier MONNET[(1)]

(1) AP-HP, Service de Médecine Intensive-Réanimation, Hôpital de Bicêtre, DMU 4 CORREVE, Inserm UMR S_999, FHU SEPSIS, CARMAS, Université Paris-Saclay, 78 rue du Général Leclerc, 94270, Le Kremlin-Bicêtre, France.

(2) Service de Médecine Intensive Réanimation, Centre Hospitalier Universitaire Grenoble Alpes, Av. des Maquis du Grésivaudan, 38700, La Tronche, France.

(3) Division of Pulmonary, Critical Care, Allergy and Sleep Medicine, Department of Medicine, Emory University School of Medicine, Atlanta, Georgia, United States.

(4) Laboratoire DAVID, Université Versailles Saint-Quentin-en-Yvelines, 78035 Versailles, France

SUPPLEMENTARY MATERIALS

A) EXPLAINABILITY VS INTERPRETABILITY DEFINITIONS:

Various definitions of explainability can be found within the AI literature, sometimes used interchangeably with the concept of interpretability. However, nuanced distinctions exist between these two notions, which are elaborated upon below.

Explainability

Explainability refers to the model's capacity to offer explanations for its decisions in a manner understandable to humans. Consequently, Explainable AI (XAI) focuses on elucidating the "why" aspect of AI-assisted decision-making, with the aim of enhancing transparency and comprehensibility for humans (1).

Considering the example of a hypothetical machine learning model predicting the risk of sepsis in critically ill patients in an ICU, explainability would involve providing a clear and understandable explanation for why the machine learning model predicted a high risk of sepsis for a specific patient. The explanation would highlight the specific clinical parameters that contributed to the high-risk prediction (e.g., elevated heart rate, abnormal white blood cell count, and low blood pressure, known factors associated with sepsis development) and provide a logical connection between these parameters and the prediction of sepsis risk. This would enable the medical team to comprehend the model's decision-making process and offer valuable insights for further evaluation and intervention.

Interpretability

On the other hand, interpretability emphasises the "how " aspect of decision-making. It refers to the ability of a learning model to reveal the process it used to reach a decision, showing the rationale behind its choices.

Continuing with the previous scenario, interpretability would involve detailing the step-by-step process by which the machine learning model derived its prediction of high sepsis risk for a specific patient. This entails delving into the specific computations, feature importance, and contributions of individual clinical indicators that influenced the model's prediction.

Some models inherently exhibit interpretability (transparent models or "white-box"), while others are not immediately understandable ("black-box"), necessitating supplementation with an interpretable and faithful explanation (post-hoc explanation). These explanations can be either global or local, applicable specifically to a particular model or universally to other models (agnostic methods).

Interpretable systems are considered explainable for a targeted audience when their processes are readily comprehensible to that audience. Conversely, one does not necessarily need to understand the mechanistic "how " to discern clear patterns in certain variables that might elucidate a model’s predictions for clinicians.

B) <u>LOCAL VERSUS GLOBAL EXPLANATION:</u>

Explanations within the domain of XAI are categorised into two main types: global and local (2).

Global Explanation: "Why does the model make its decisions in the way that it does, in general?"

Global explanations delve into the model's decision-making process at a macro level, offering insights into its foundational mechanisms and overarching strategies, such as identifying the most influential features for a given predictive task.

Local Explanation: "Why did the model make this particular decision for this specific instance?"

Local explanations narrow the focus to the justification of individual decisions, shedding light on the specific reasoning behind distinct predictions for particular instances.

C) <u>EXPLAINABILITY METHODS TAXONOMY:</u>

Here we present a classification of explainability methods, accompanied by examples for each category. Extended descriptions and implementation examples can be found elsewhere (3).

I. DATA EXPLAINABILITY TECHNIQUES:

a) **Exploratory Analysis and Visualisation:**

Initial techniques to understand the data's patterns, distributions, and relationships.

- **Histograms and Bar Charts, Scatter Plots, Heat Maps…**

b) Dimensionality Reduction techniques:

Essential for making high-dimensional data interpretable. Here are examples of most used techniques:

- **PCA (Principal Component Analysis):**

Transforms correlated features into uncorrelated ones, capturing the most variance with fewer dimensions. It uses orthogonal transformations and is best suited for linear relationships.

● **t-SNE (t-Distributed Stochastic Neighbour Embedding):**

Visualises high-dimensional data by preserving neighbouring point structures. It's effective for visualising clusters, especially with non-linear relationships, but results may vary on repeated runs.

II. INTRINSICALLY INTERPRETABLE / WHITE-BOX MODELS:

Models where the decision-making process is inherently transparent.

● **Linear Regression/Logistic Regression**:

Feature coefficients indicate importance and influence direction. Direct inspection of coefficients reveals feature contributions.

● **Decision Trees:**

Tree structures where nodes represent decisions based on feature values. The tree can be visualised, and feature depth indicates importance.

● **Generalised Additive Models (GAMs):**

Extensions of linear models that capture non-linear relationships using smooth functions. Each feature has its own function, visualised to understand its relationship with the output.

III. POST-HOC APPROACHES:

Methods that explain decisions after model training, categorised into model-specific or model-agnostic.

1. Model-agnostic strategies:

Model-agnostic strategies are universally applicable, analysing feature-output relationships and data distributions without regard to the specific model's internal workings.

a) Surrogate Models:

Surrogate models leverage simpler, more transparent algorithms like linear regression or decision trees to approximate the outputs of complex AI models. These models facilitate a deeper understanding of the intricate model's logic and decision-making process.

• **Global Surrogate Models:**

Utilising data that mirrors the distribution and characteristics of the original model's training set, these models employ intuitive methods such as Generalised Additive Models (GAM), Logistic Regression, or Decision Trees. Their effectiveness lies in their ability to broadly replicate the complex model's outputs, offering a window into its overall operation and logic.

• **LIME (Local Interpretable Model-agnostic Explanations):**

Works by perturbing the input data and observing how the predictions change. For example, if the input is an image, LIME modifies small regions of the image, such as turning superpixels (connected groups of pixels with similar colours) on or off. It then fits a local surrogate model to approximate the complex model's behaviour, providing insights into how the model

behaves near specific instances (e.g., a particular image). As a Local Surrogate Model, it is designed for explaining 'individual' predictions.

b) Visualisation Techniques:

• Partial Dependence Plots (PDP):

PDPs visualise the effect of a single feature on the predicted outcome, averaged over all other features. They help in understanding whether the relationship between the target and a feature is linear, monotonic, or more complex.

c) Feature-based Explanations:

These techniques focus on the contribution and importance of individual input features to the model's output.

● Permutation Importance:

By randomly shuffling each predictor one at a time and observing the degradation in the model's performance, this method gauges the importance of each feature. It's a robust method but can be computationally intensive.

● SHAP (SHapley Additive exPlanations):

SHAP (SHapley Additive exPlanations) is a method from game theory used to explain machine learning model predictions. Each feature (e.g. age, blood pressure, cholesterol level..) is treated as a "player" in a game, with the prediction being the "payout." SHAP values measure the average contribution of each feature by evaluating how the prediction changes when the feature is included in all possible combinations of features. SHAP provides a unified measure

of feature importance and offers both local (individual predictions) and global (overall behaviour) explainability.

d) Counterfactual Explanations:

Counterfactual explanations describe hypothetical scenarios that illustrate how altering specific inputs can change a model's prediction. For example, if a model predicts a high risk of sepsis based on certain vital signs, a counterfactual explanation might state: "If the white blood cell count were lower, the predicted risk of sepsis would decrease." This approach highlights the causal impact of particular features on the model's outcome. Counterfactual explanations are especially human-friendly because they are contrastive, focusing on differences from the current scenario, and selective, typically involving minimal changes to the inputs. However, one situation can generate multiple counterfactuals, leading to the "Rashomon effect," where different, yet plausible, explanations exist for the same outcome. This multiplicity requires careful consideration in selecting the most relevant or useful counterfactual explanation.

2. Model-specific approaches:

Model-specific approaches delve deeply into a model's architecture and processes, focusing on the Algorithm’s structure and intermediate stages, but their application is limited to certain types of algorithms.

- **Visual Explanations (Saliency Mapping):**

Saliency mapping is primarily used in neural networks, particularly for tasks like image classification. It visually highlights regions of an input image that are crucial for a model's

decision by computing saliency scores. These scores measure the contribution of individual pixels to the network's output. By creating a saliency map, this technique identifies which parts of the image the model focuses on during its decision-making process.

Supplementary Fig 1. A Taxonomy of XAI Methods

As noted by Speith et al. (4), *there is no universally agreed-upon taxonomy, reflecting the extensive scope and dynamic evolution of the field, as well as the diverse goals of various stakeholders in AI explainability.*

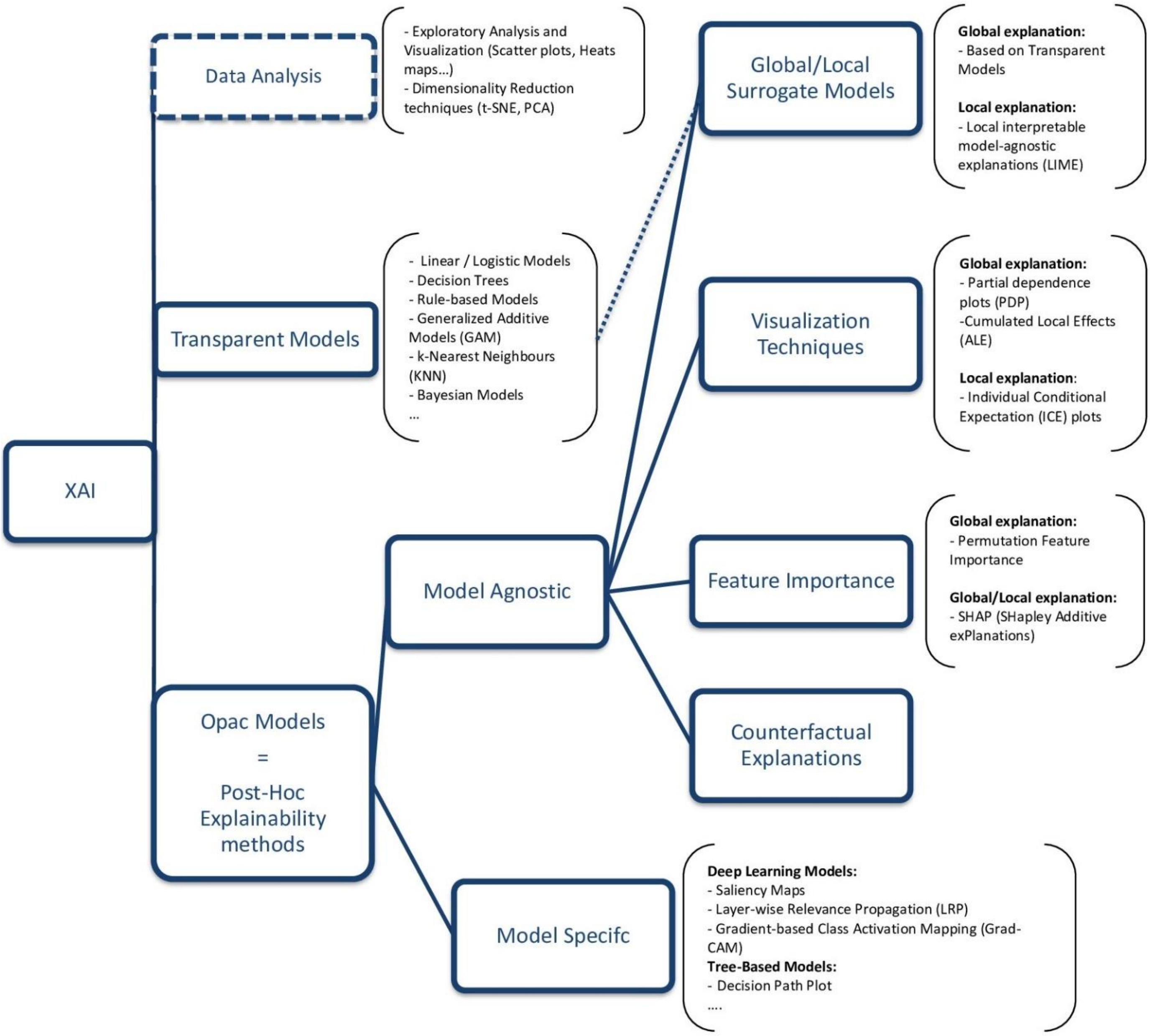

Supplementary Fig. 2 Classical evoked balance between the need for justify individual decision (e.g. in life-threatening medical situations), and model performance.

The prevailing notion of a trade-off between accuracy and explainability is now being questioned, especially in medical models with detailed, structured data, rooted in physiopathology. In these cases, the disparity in performance between interpretable and more complex models is often negligible (5).

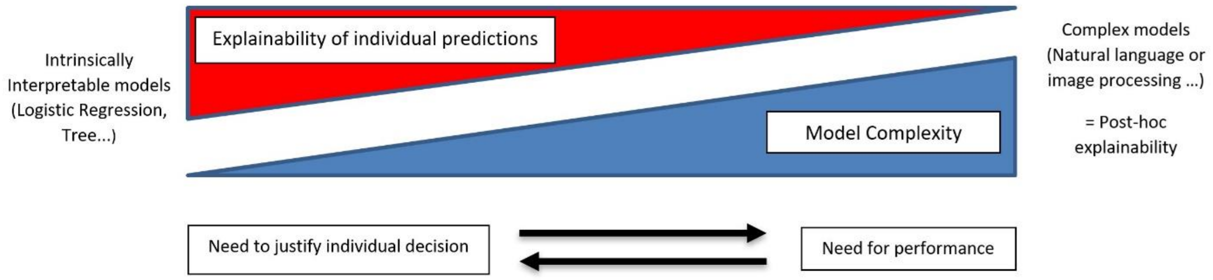

References:

1. Bharati S, Mondal MRH, Podder P. A Review on Explainable Artificial Intelligence for Healthcare: Why, How, and When? IEEE Trans Artif Intell. 2023;1–15.

2. Van Der Velden BHM, Kuijf HJ, Gilhuijs KGA, Viergever MA. Explainable artificial intelligence (XAI) in deep learning-based medical image analysis. Medical Image Analysis. 2022 Jul;79:102470.

3. Molnar C. Interpretable machine learning: a guide for making black box models explainable. Second edition. Munich, Germany: Christoph Molnar; 2022. 318 p.

4. Speith T. A Review of Taxonomies of Explainable Artificial Intelligence (XAI) Methods. In: 2022 ACM Conference on Fairness, Accountability, and Transparency [Internet]. Seoul Republic of Korea: ACM; 2022 [cited 2024 Mar 11]. p. 2239–50. Available from: https://dl.acm.org/doi/10.1145/3531146.3534639

5. Rudin C. Stop explaining black box machine learning models for high stakes decisions and use interpretable models instead. Nat Mach Intell. 2019 May 13;1(5):206–15.
